# Combined prevalence of impaired glucose level or diabetes and its correlates in Lusaka urban district, Zambia: a population based survey

**DOI:** 10.1186/1755-7682-4-2

**Published:** 2011-01-12

**Authors:** Mutale Nsakashalo-Senkwe, Seter Siziya, Fastone M Goma, Peter Songolo, Victor Mukonka, Olusegun Babaniyi

**Affiliations:** 1 Directorate of Public Health and Research, Ministry of Health, Lusaka, Zambia; 2 Department of Community Medicine, School of Medicine, University of Zambia, Lusaka, Zambia; 3 Department of Physiology, School of Medicine, University of Zambia, Lusaka, Zambia; 4 World Health Organization, Country Office, Lusaka, Zambia

## Abstract

**Background:**

Developing countries are undergoing an epidemiological transition, from Communicable or Infectious to 'Non-Communicable' diseases (NCDs), such that cardiovascular disease, chronic respiratory diseases, cancer, and diabetes were responsible for 60% of all deaths globally in 2005, with more than 75% of these deaths occurring in developing countries. A survey was conducted to determine among other objectives the prevalence of diabetes and its association with physical fitness and biological factors.

**Methods:**

A cross sectional study utilizing a modified World Health Organization's STEPwise approach to surveillance of NCDs was conducted in Lusaka district, Zambia. A multi-stage cluster sampling technique was used to select study participants of age 25 years or older. All eligible members of a household that was selected were invited to participate in the study. Unadjusted odds ratios (OR), and adjusted odds ratios (AOR) together with their 95% Confidence Intervals (CI) were obtained using Complex samples logistic regression

**Results:**

A total of 1928 individuals participated in the survey, of which 33.0% were males. About half of the participants were of age 25-34 years (53.2%), and about a third of the respondents had attained secondary level of education (35.8%). The combined prevalence for impaired glucose level or diabetes was 4.0%. Age and mild hypertension were significantly associated with impaired levels of glucose or diabetes. Compared to participants in the age group 25-34 years, older participants were more likely to have impaired glucose level or diabetes (AOR = 2.49 (95%CI [1.35, 2.92]) for 35-44 years age group, and AOR = 3.80 (95%CI [2.00, 7.23]) for 45 + years age group). Mild hypertension was associated with impaired glucose level or diabetes (AOR = 2.57) (95%CI [1.44, 4.57])).

**Conclusions:**

The prevalence of diabetes in Lusaka district has not reached an alarming level and it is now that interventions targeting the younger age group 25-34 years should be put in place to curtail the spread of diabetes.

## Background

The major Non-Communicable Diseases (NCDs) that include diabetes contribute immensely to mortality [1]. All the NCDs are associated with identifiable behavioural risk factors and biological risk factors. These two groups of risk factors are closely linked. The major behavioural risk factors are tobacco use, unhealthy diet and physical inactivity [2]. And the major biological risk factors include; obesity, hypertension, diabetes and dyslipidemia [3]; and genetic predisposition mainly accounting for type I diabetes. Most of these factors are modifiable through lifestyle interventions.

Work and living situations have become more sedentary thus increasing the risk of NCDs [2]. Physical inactivity increases the risk of many chronic diseases, such as type 2 diabetes [4,5]. Metabolic syndrome which is a group of disorders that include obesity, insulin resistance, glucose intolerance, abnormal lipids and hypertension has been associated with reduced physical activities [6,7]. Low physical activity like prolonged television viewing may contribute to metabolic syndrome through related poor eating habits [4]. Several studies have showed an association between prolonged television viewing and metabolic syndrome [4,6]. Metabolic syndrome has been linked to type 2 *Diabetes mellitus*, cardiovascular diseases and mortality and therefore reducing sedentary behaviour has a role in the prevention of these chronic diseases [4]. Nelson and Gordon-Larsen [7] observed that enhancing opportunities for increased exercises and sport may have a beneficial effect in modulating risk behaviours in the adolescent population.

The control of NCDs including diabetes has received little attention. For instance, the reduction of Non Communicable diseases is not a Millennium Development Goal. Many governments and organisations have focussed on controlling diseases like HIV/AIDS, malaria and Tuberculosis, and neglecting NCDs [8].

In 2004, 33.1% of male and 32.7% of female school going adolescents of age 13-15 years in Zambia reported spending three or more hours during a typical day sitting and watching television, playing computer games, talking with friends, or doing other sitting activities [9]. No similar studies have been conducted among non-school going adolescents or among older age groups. A survey was conducted to determine among other objectives the combined prevalence of impaired glucose level or diabetes and its association with physical fitness and biological factors.

## Methods

The study was conducted in Lusaka district in low, medium and high density residential areas. A cross sectional study utilising a modified WHO global surveillance initiative NCD-STEP 3 [10] was used in the current study.

### Sample size

In a study whose results were designed to reflect national estimates, a total sample size of 6128 respondents was calculated for the entire country. The sample size was powered enough to produce estimates for rural/urban comparisons, and for between gender and districts. Lusaka district sample size was 1915 participants.

### Sampling

A multi-stage cluster sampling technique was used to select study participants. Lusaka province being the most urbanised province in Zambia was conveniently sampled. In the second stage of sampling, only Lusaka urban district was conveniently selected from the three districts in Lusaka (the other two being Kafue that is a peri-urban district; and Chongwe that is a rural district). Lusaka district had 7 constituencies out of which 5 were randomly selected. From each selected constituency, one ward was selected. The number of Standard Enumeration Areas (SEAs) selected in each ward was proportional to its population size. SEAs were selected using a systematic random sampling method. Households were then systematically sampled in order to widely cover the selected SEAs. All persons of ages 25 or more years were invited to participate in the survey.

### Ethical considerations

The study protocol was reviewed by the University of Zambia (UNZA) Research Ethics Committee (REC), and the study only commenced when approval from the UNZA REC was granted. All entry forms were kept in the office of the Principal Investigator. Entry forms were only viewed by approved study personnel.

### Data collection

The WHO global surveillance initiative for NCD (World Health Organization, 2005b) has three steps: Step 1 is the questionnaire, Step 2 is physical examinations, and Step 3 is biochemical examinations. All these steps were conducted within the participants' houses.

#### Interviews

An interview schedule was used to elicit responses from the interviewees. The questionnaire was divided into the following sections among others: Demographic information, Alcohol consumption, Sedentary behaviour (time usually spent sitting or reclining on a typical day), Physical measurements (Height and Weight, Waist, Blood pressure, and Hip circumference) and Biochemical measurements (Blood glucose, and HDL cholesterol). Interviews were conducted in the homes of the participants.

#### Measurements

The WHO STEPs surveillance training and practical guide recommends that physical measurements be taken in the following order: height, weight, waist circumference, and blood pressure. We chose to take blood pressure readings first, after having administered the questionnaire. This gave the participant enough time to have settled down.

#### Blood pressure

The Omron Digital Automatic BP Monitor M4-1 was used to measure the blood pressure of the participants. Three minutes of rest was given to the participant in between three successive readings of blood pressure. Although the three readings were different with the largest value being the first reading and the smallest being the third reading on average, these differed by no more than 2 mm/Hg of systolic blood pressure, and no more than 4.5 mm/Hg of diastolic blood pressure. We chose to take an average of the three reading, and not the average of the second and third readings as recommended by World Health Organisation in order to increase the degrees of freedom for the mean.

Systolic blood pressure (SBP) was grouped into four levels: <140 (normal), 140-169 (mildly raised), 170-179 (moderately raised), and 180+ (severely raised); similarly diastolic blood pressure (DBP) was grouped into four levels: <90 (normal), 90-99 (mildly raised), 100-109 (moderately raised), and 110+ (severely raised). A participant was classified as having normal hypertension if SBP and DBP were both normal; Meanwhile, a participant was classified as having mild hypertension when SBP or DBP was mildly raised; classified as moderate hypertension when SBP or DBP was moderately raised; and was classified as severe hypertension when SBP or DBP was severely raised. In this study we did not consider participants who were on treatment for hypertension or were previously diagnosed as being hypertensive since we sorely relied on the current SBP or BDP readings. Known cases of hypertension may alter their behaviour depending on their current status of hypertension; and it is for this reason we classified participants as we did.

#### Height

The Seca Brand 214 Portable Stadiometer was used to measure the heights of the participants. Height was measured without the participants wearing foot or head gear. Before the reading was taken, the participants were requested to have their feet together, heels against the back board, knees straight, and look straight ahead. Height was recorded in centimetres.

#### Weight

Weight was measured using the Heine Portable Professional Adult Scale 737. Participants were asked to stand still, face forward, and place arms on the sides of the body. Weight was recorded in kilograms.

#### Waist circumference

The Figure Finder Tape Measure was used to measure the waist circumference in centimetres. This measurement was taken in a private area. The midpoint between the inferior margin of the last rib and the crest of the ilium were marked using a tape measure. With the assistance of the participants, the tape measure was wrapped around the waist directly over the skin or light clothing. Just before the measurement was taken, participants were requested to stand with their feet together, place their arms at their side of their body with the palms of their hands facing inwards, and breathe out gently.

#### Hip circumference

The measurement for hip circumference was taken in a private area immediately after the waist circumference. The Figure Finder tape Measure was used in measuring the hip circumference in centimetres. The measurement was taken at the maximum circumference over the buttocks, after requesting the participants to relax their arms at the sides.

#### Biochemical measurements

Fasting glucose and total cholesterol were determined using an Accutrend GCT Meter Three-in-One System for Glucose, Cholesterol and Triglycerides. Glucose levels were grouped into low glucose levels (<3.3 mmol/L), normal (3.3-5.5 mmol/L), impaired glucose level (5.51-8.49 mmol/L), and diabetes (8.5 mmol/L or more); and cholesterol levels were either normal (<5.2 mmol/L) or otherwise raised.

### Data entry

Two data entry clerks were trained to enter the data using Epi Data version 3.1. Data was double entered and validated. The data entry template had consistency and range checks embedded in it. The data entry clerks were trained and supervised by SS. The validated data was exported to SPSS version 14.0 for analysis.

### Data analysis

Body Mass Index (BMI) was categorized as <18.5 (underweight), 18.5-24.9 (normal), 25.0-29.9 (over weight), and 30+ (obese); and waist-hip ratios was grouped into two: ≤1 (normal) and >1 (raised). The analysis included running frequencies, cross-tabulations, bivariate, and multivariate Complex samples logistic regression. Unadjusted odds ratios (OR), and adjusted odds ratios (AOR) together with their 95%CI were computed.

## Results

A total of 1928 individuals participated in the survey, of which 33.0% were males. About half of the participants were of age 25-34 years (53.2%), and a third of the respondents had attained secondary level of education (35.8%). About 1 in 5 of the respondents were either self employed (22.5%) or housewives (20.0%). Further description of the sample is presented in Table 1.

**Table 1:** Demographic characteristics for the sampled population.

	Total	Male	Female
Variable	n (%)	n (%)	n (%)
Age group (years)			
25-34	1015 (53.2)	337 (53.7)	675 (52.9)
35-44	413 (21.6)	135 (21.5)	277 (21.7)
45+	481 (25.2)	156 (24.8)	323 (25.3)
			
Sex			
Male	634 (33.0)	-	-
Female	1288 (67.0)	-	-
			
Education			
None	408 (21.5)	76 (12.2)	330 (26.0)
Primary	276 (14.5)	61 (9.8)	214 (16.9)
Secondary	679 (35.8)	242 (38.8)	435 (34.3)
College/university	534 (28.1)	244 (39.2)	290 (22.9)

NB: numbers do not add up due to missing information

### Impaired glucose level or diabetes

Of the total of 1880 subjects who had fasting blood sugar measurements done, 24 (1.3%) had impaired glucose level (8 males (33.3%) and 16 females (66.7%) while 51 (2.7%) had diabetes (13 (25.5%) males and 38 (74.5%) females). The age by sex standardised rates for combined impaired glucose level or diabetes for males and females considering Lusaka's age by sex population were 4.9% for males and 5.6% for females.

Table 2 shows the distribution of the combined prevalence of impaired glucose or diabetes by risk factors considered in the current study. The prevalence varied with age with the highest prevalence being in the age group 45+ years. The highest prevalence was reported among participants who were obese. Participants with raised cholesterol had a higher prevalence than those with normal levels of cholesterol. No clear pattern emerged for the prevalence among levels of hypertension.

**Table 2:** Distribution of combined prevalence of impaired glucose level or diabetes by risk factors considered in the current study.

Variable	Total	n (%)	p value
Age group (years)			<0.001
25-34	748	9 (1.2)	
35-44	329	19 (5.8)	
45+	390	46 (11.8)	
			
Sex			0.364
Male	495	21 (4.2)	
Female	983	54 (5.5)	
Education			0.304
			
None	315	18 (5.7)	
Primary	218	15 (6.9)	
Secondary	507	19 (3.7)	
College/university	417	22 (5.3)	
			
Body Mass Index (kg/m^2^)			<0.001
<18.5	105	5 (4.8)	
18.5-24.9	754	18 (2.4)	
25.0-29.9	385	22 (5.7)	
30+	222	30 (13.5)	
			
Time usually spent sitting or reclining on a typical day (hours) (sedentary behaviour)			0.829
<1.5	359	19 (5.3)	
1.5-3.4	616	28 (4.5)	
3.5+	499	26 (5.20	
			
Currently consumed alcohol			1.000
Yes	384	19 (4.9)	
No	1095	55 (5.0)	
			
Waist Hip ratio			0.001
≤ 1	1410	70 (5.0)	
> 1	15	10 (*)	
			
Hypertension			<0.001
Normal	939	21 (2.2)	
Mild	330	33 (10.0)	
Moderate	89	5 (5.6)	
Severe	97	13 (13.4)	
			
Cholesterol			0.036
Normal	1234	54 (4.4)	
Raised	243	19 (7.8)	

NB: numbers do not add up due to missing information

* Denominator less than 30 and cannot compute the percent

Percents are variable specific

Factors associated with combined impaired levels of glucose or diabetes are presented in Table 3. Age, body mass index, waist-hip ratio, hypertension, and cholesterol were significantly associated with combined impaired glucose level or diabetes in bivariate analyses. In multivariate analysis, compared to participants in the age group 25-34 years, older participants were more likely to have combined impaired glucose level or diabetes (AOR = 2.49 (95%CI [1.35, 2.92]) for 35-44 years age group, and AOR = 3.80 (95%CI [2.00, 7.23]) for 45+ years age group). Only mild hypertension was associated with combined impaired glucose level or diabetes (AOR = 2.57 (95%CI [1.44, 4.57])). No significant associations were observed between moderate or severe hypertension and combined impaired glucose level or diabetes.

**Table 3:** Factors associated with combined impaired glucose level or diabetes

	Unadjusted	Adjusted
Variable	OR (95%CI)	AOR (95%CI)
Age group (years)		
25-34	1	1
35-44	5.03 (2.25, 11.25)	2.49 (1.26, 4.92)
45+	10.98 (5.33, 22.64)	3.80 (2.00, 7.23)
		
Sex		
Male	1	-
Female	1.31 (0.78, 2.20)	
		
Education		
None	1	-
Primary	1.22 (0.60, 2.48)	
Secondary	0.64 (0.33, 1.25)	
College/university	0.92 (0.48, 1.75)	
		
Body Mass Index (kg/m^2^)		
<18.5	1	1
18.5-24.9	0.49 (0.18, 1.35)	0.51 (0.20, 1.35)
25.0-29.9	1.21 (0.45, 3.29)	0.82 (0.30, 2.25)
30+	3.13 (1.18, 8.31)	1.76 (0.64, 4.87)
		
Time usually spent sitting or reclining on a typical day (hours) (sedentary behaviour)		
<1.5	1	-
1.5-3.4	0.85 (0.47, 1.55)	
3.5+	0.98 (0.54, 1.81)	
Currently consumed alcohol		
Yes	1	-
No	1.54 (0.53, 4.43)	
		
Waist Hip ratio		
≤ 1	1	*
> 1	9.57 (3.18, 28.83)	
		
Hypertension		
Normal	1	1
Mild	4.86 (2.76, 8.54)	2.57 (1.44, 4.57)
Moderate	2.60 (0.96, 7.09)	0.98 (0.34, 2.83)
Severe	6.77 (3.26, 14.02)	1.84 (0.75, 4.49)
		
Cholesterol		
Normal	1	1
Raised	1.85 (1.08, 3.19)	1.23 (0.67, 2.26)

* not enough data in one cell to compute the estimate and its confidence interval

## Discussion

This is the first study to report results from a comprehensive general population-based survey on the prevalence rate of impaired glucose level or diabetes and its correlates among persons of age 25 years or more in Lusaka urban district. The findings in the current study form baseline information to which interventions to control diabetes in Lusaka urban district could be measured against.

We found that the prevalence of diabetes was 2.1% among males and 3.0% among females in the present study, and that of impaired glucose level was 1.3% and 1.3% for males and females, respectively. The prevalence rates of diabetes in Nauru of 9.4% among 25-64 years olds [11], in Northern Sudan of 9.9% among males and 7.5% among females [12], and in South Africa among elderly, the prevalence of diabetes of 25.7% among males and 30.3% among females, and that of impaired glucose tolerance of 15% overall [13] were much higher than what we found in Lusaka urban district, Zambia. However, our rates are comparable to what was found in Ho Chi Minh City (HCMC) of 2.7% for men and 2.6% for women [14], and in Zimbabwe of 2.9% males and 2.3% females known to have diabetes mellitus [15]. A possible explanation for these differences lies in the differences in the age groups of the participants, and in the definitions of diabetes. For example our age group was 25 years or older while in the South African study the age group of the participants was 65 years or older. Raised blood glucose was defined as capillary whole blood glucose of at least 6.1 mmol/L in the HCMC study, while our cut off point for impaired glucose level was 5.51 mmol/L, and that for diabetes was 8.5 mmol/L.

We found an alarmingly high rate of about 1 in 5 participants with low glucose levels. We calibrated the meters by testing the glucose levels for known diabetic and non-diabetic persons; and the inaccuracy of the meter may not have been the source of this data. Hence, the high prevalence may be a true reflection of the status of the study population. This observation calls for further studies to elucidate this finding.

Only 0.2% of males reported seeing a traditional healer for diabetes in the current study, This figure may be an underestimate because many diabetic patients in Africa expect that the disease can be cured, not just controlled, and they turn preferentially to traditional healers who promise cures, rather than to conventional modern medicine [16].

In our study, we found that diabetes or impaired glucose level was associated with older age groups. Similarly, Elbagir et al [17] reported that age was significantly associated with higher rates of diabetes. In 1998 these authors also reported a significant association between age and diabetes [12]. Among the Hindu Indian immigrants in Dar es Salaam, age was one of the major risk factors for diabetes [18]. A similar finding of an association between age and diabetes was reported among women in the Mekong Delta, Vietnam [19].

In our study, we found that mild hypertension was associated with combined impaired glucose level or diabetes. The inconsistent finding that moderate or severe hypertension was not significantly associated with combined impaired glucose level or diabetes may partly be explained by the small sample sizes for these levels of hypertension.

### Possible limitations

Though the study design provides reliable and valid information, the study may have some limitations. The survey was done in Lusaka urban district, and hence the results can only be generalized to the sampled population. We did not have reliable information on the number of household members of age 25 years or older in order to enable us to compute response rates. Therefore, we could not compute weights that could have been used in the analysis. Our findings may be biased to the extent that non-respondents differed from those that participated in the survey. However, we are unable to suggest the direction of bias. Some information on the important risk factors for diabetes was either not collected or inadequately collected (such as family history of diabetes and diet). We acknowledge lack of this information as a limitation to the study that may have confounded our findings. Some study factors in our survey were obtained through self-reports, and as in all such studies, both inadvertent and deliberate reporting is a concern, more so that we obtained personal identifiers. In spite of the above limitations, we believe that our findings are credible as they compare favourably with those obtained in the Zambia Demographic and Health Survey.

## Conclusions

The prevalence of diabetes in Lusaka district has not reached an alarming level and it is now that interventions targeting the age groups 25-34 years should be put in place to curtail the spread of diabetes.

## Competing interests

The authors declare that they have no competing interests.

## Authors' contributions

MN-S contributed to the interpretation of the findings; SS contributed to the design of the study, training of research assistants, data acquisition, data analysis and interpretation of the findings, and led the drafting of the manuscript; FMG contributed to training of interviewers, data acquisition, and interpretation of the findings; PS contributed to the design of the study and interpretation of the findings; VM contributed to the design of the study and interpretation of the findings; OB contributed to the interpretation of the findings; All authors read and approved the final version of the document.
